# Epidemiology and secular trends of malignant lymphoma in Japan: Analysis of 9426 cases according to the World Health Organization classification

**DOI:** 10.1002/cam4.1805

**Published:** 2018-10-11

**Authors:** Reiji Muto, Hiroaki Miyoshi, Kensaku Sato, Takuya Furuta, Hiroko Muta, Keisuke Kawamoto, Eriko Yanagida, Kyohei Yamada, Koichi Ohshima

**Affiliations:** ^1^ Department of Pathology Kurume University School of Medicine Kurume Japan; ^2^ Department of Pathology, National Hospital Organization Kumamoto Medical Center Kumamoto Japan; ^3^ Faculty of Medicine, Hematology, Endocrinology, and Metabolism Niigata University Niigata Niigata Japan

**Keywords:** adult T‐cell leukemia/lymphoma, diffuse large B‐cell lymphoma, epidemiology, follicular lymphoma, Japan

## Abstract

This study provides an overview of the epidemiology and secular trends of malignant lymphoma in Japan. Using data from clinics and hospitals throughout Japan, we analyzed 9426 cases of malignant lymphoma diagnosed in 2007‐2014. We show that the proportion of follicular lymphoma and methotrexate‐associated lymphoproliferative disorder increased during this time, as did the onset age for follicular lymphoma and diffuse large B‐cell lymphoma. Significant increases in onset age for follicular lymphoma and diffuse large B‐cell lymphoma were observed in both men and women (all *P* values <0.0001 except for *P* = 0.0448 for the latter disease in women). Further studies are required to determine the reasons for the higher proportion of and onset age for these lymphomas. Additionally, we believe that continued observation of these trends is necessary.

## 1 INTRODUCTION

The epidemiological characteristics of malignant lymphoma (eg, proportion, sex ratio, and age of onset) differ along sociological, environmental, and racial lines.^1^, ^2^, ^3^, ^4^

In adult B‐cell lymphoma, diffuse large B‐cell lymphoma (DLBCL, 37%) and follicular lymphoma (FL, 29%) are the most common subtypes worldwide, followed by extranodal marginal zone lymphoma of mucosa‐associated lymphoid tissue (MALT, 9%), mantle cell lymphoma (MCL, 7%), and chronic lymphoid leukemia/small lymphoid leukemia (CLL/SLL, 12%).^5^ However, these proportions vary from country to country. CLL/SLL, for example, is less common in Asia than in other parts of the world.^5^

In adult T‐cell lymphoma, peripheral T‐cell lymphoma not otherwise specified (PTCL‐NOS) and angioimmunoblastic T‐cell lymphoma (AITL) are the most common subtypes worldwide.^6^ According to the international peripheral T‐cell and natural killer (NK)/T‐cell lymphoma study, their relative frequencies are 25.9% and 18.5%, respectively.^6^ Other common T‐cell lymphoma subtypes include extranodal NK/T‐cell lymphoma (10.4%), adult T‐cell leukemia/lymphoma (ATLL, 9.6%), anaplastic lymphoma kinase‐positive anaplastic large cell lymphoma (ALCL‐ALK^＋^, 9.6%), and ALK‐negative (ALCL‐ALK^−^, 5.5%).^6^ These proportions vary according to geographic locations; extranodal NK/T‐cell lymphoma and ATLL, for example, are more common in Asia than in Europe and the USA.^5^

Epidemiological studies of malignant lymphoma in Japan have been performed by the Lymphoma Study Group of Japanese Pathologists (3194 cases in 2000)^7^ and Aoki et al (2260 cases in 2008).^8^ The latter study examined geographic distribution, age of onset, and sex ratio in cases diagnosed at Kurume University from 2001 to 2006 using data from clinics and hospitals throughout Japan. However, no paper on the secular trend of epidemiological factors in Japan has been published to date.

Herein, we present an overview of the epidemiology and secular trends of malignant lymphomas diagnosed from 2007 to 2014 in Japan.

## 2 MATERIALS AND METHODS

### 2.1 Tissue samples

We reviewed 19 332 clinically suspected cases of hematopoietic/lymphoid tissue malignancies that were diagnosed in our laboratory between 2007 and 2014. For diagnosis, biopsy or resection specimens were sent to us from 396 hospitals and clinics across Japan's 47 prefectures, ranging from the northernmost prefecture of Hokkaido to Okinawa, the southernmost prefecture. Clinical data were obtained from clinicians at the time of the initial diagnosis.

Histological confirmation was obtained in 11 428 of the 19 332 cases, with 9426 remaining after exclusion of cases with relapses or recurrences. Malignant lymphomas were diagnosed and analyzed in accordance with the 2008 World Health Organization (WHO) guidelines. We used three procedures (described below) for diagnosis: flow cytometry, immunohistochemistry, and DNA and RNA analysis. All final diagnoses were made by one pathologist (K. O.), who is an authority on the pathology of lymphoid neoplasms. This study was approved by the Research Ethics Committee of Kurume University.

### 2.2 Flow cytometry

Antibodies (in parentheses) to the following proteins were used for flow cytometry: CD45 (2D1), CD2 (T11:SFCI3Pt2H9), CD3 (UCHT1), CD4 (T4:SFCI12T4D11), CD5 (T1:SFCI24T6G12), CD7 (3A1:3A1E‐12H7), CD8 (T8:SFCI21Thy2D3), CD10 (J5), CD11c (BU15), CD16 (3G8), CD19 (B4:89B), CD20 (L27), CD23 (MHM6), CD25(2A3), CD30 (Ber‐H2), CD34 (581), CD56 (NHK‐1:N901), κ‐chain, and λ‐chain. All antibodies were from Beckman Coulter Inc (Brea, CA, USA), except for those to CD45, CD20, and CD45 (Becton Dickinson Biosciences, Tokyo, Japan) and CD23, CD30, κ chain, and λ chain (Agilent Technologies, Santa Clara, CA, USA). Isotype control antibodies (MsIg‐FITC and MsIg‐RD1) were obtained from Beckman Coulter Inc.

### 2.3 Immunohistochemistry

For immunohistochemistry, formalin‐fixed paraffin‐embedded tissue and/or frozen sections were used. All tissues and sections were stained with H&E and immunostained for pan‐B‐cell (CD20) and pan‐T‐cell (CD45RO) markers. Ancillary immunostaining was performed when needed. Antibodies (in parentheses) to the following proteins or cells were used for immunohistochemistry: CD1a (EP3622), CD3 (2GV6), CD4 (SP35), CD5 (SP19), CD8 (SP57), CD10 (SP67), CD21 (2G9), CD23 (SP23), CD30 (Ber‐H2), CD34 (QBEnd/10), CD56 (MRQ‐42), CD79a (SP18), CD138 (B‐A38), cyclin D1 (SP4‐R), bcl2 (SP66), bcl6 (GI191E/A8), multiple myeloma oncogene 1 (MRQ43), myeloperoxidase (polyclonal), terminal deoxyribonucleotidyl transferase (polyclonal), cytokeratin AE1/AE3 (AE1/AE3/PCK26), and ALK (ALK01) (Roche Diagnostics, Basel, Switzerland); CD7 (LP15), CD13 (38C12), CD33 (PWS44), Epstein‐Barr virus (EBV)‐encoded nuclear antigen 2 (PE2), and paired box protein 5 (1EW) (Leica Biosystems, Tokyo, Japan); CD14 (M5E2), CD15 (MMA), CD57 (HNK‐1), and CD123 (SSDCLY107D2) (Becton Dickinson Biosciences); CD19 (LE‐CD19), CD20 (L26), CD68 (PG‐M1), κ chain (polyclonal), λ chain (polyclonal), EBV‐encoded latent membrane protein (CS.1‐4), and follicular dendritic cells (CAN.42) (Agilent Technologies); chemokine (C‐X‐C motif) ligand 13 (53610) (R&D Systems, Minneapolis, MN, USA); CD25 (PC61.5), lymphoid enhancer‐binding factor 1 (EPR2029Y), programmed cell death protein 1 (NAT105), and c‐myc (Y69) (Abcam, London, UK); CD41 (P2) and T‐cell‐restricted intracellular antigen‐1 (2G9A10F5) (Beckman Coulter Inc); and epithelial membrane antigen (E29) (Nichirei Biosciences Inc, Tokyo, Japan).

### 2.4 DNA and RNA analysis

The procedures for in situ hybridization for EBV‐encoded RNA,^9^ southern blotting,^10^ and fluorescence in situ hybridization^11^ were described in detail previously. Fluorescence in situ hybridization was conducted to identify genetic abnormalities strongly associated with specific malignant lymphomas. The abnormalities examined were *IgH/BCL1* t(11;14), *IgH/BCL2* t(14;18), *BCL6* 3q27 translocation, *IgH/C‐MYC* t(8;14), *ALK* 2p23 translocation, and *API2/MALT1* t(11;18).

### 2.5 Geographical, age, and sex distributions of the histological subtypes of malignant lymphoma

For geographical distribution, we divided Japan into six regions as done by Aoki et al: Okinawa, Kyushu, Kinki/Chugoku/Shikoku, Chubu, and Kanto, Tohoku/Hokkaido.^8^ The geographical region for each case corresponded to the location of the hospital or clinic that sent the material. Okinawa and Kyushu are considered separately because both are areas in which ATLL is endemic. For age distribution, we divided the patients into nine age groups: 0‐9, 10‐19, 20‐29, 30‐39, 40‐49, 50‐59, 60‐69, 70‐79, and >80 years. Sex distributions are presented as male‐female (M/F) ratios.

### 2.6 Statistical analysis

For analysis of the secular trends of the epidemiological factors, we divided the cases into three groups based on the time of diagnosis: 2007‐2009, 2010‐2012 and 2013‐2014. Age adjustment was performed in accordance with the 1985 population model in Japan. We used Fisher's exact test to construct r × c tables. *P* values <0.05 were considered statistically significant in a two‐tailed test. Statistical analyses were conducted by using SAS 9.4 software (SAS Institute Inc, Cary, NC, USA).

## 3 RESULTS

### 3.1 Histologic subtypes

**Figure 1:**
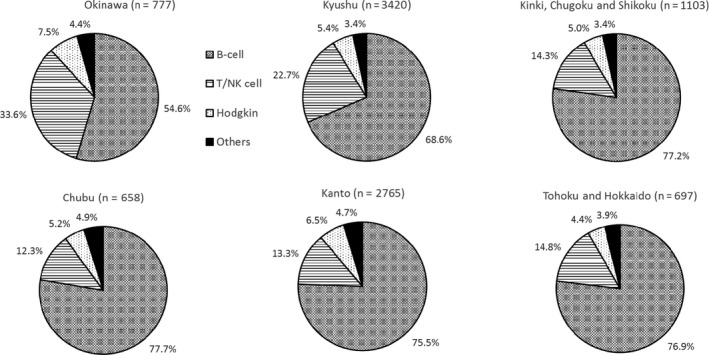
Frequency of the major histological lymphoma subtypes in six geographic areas in Japan in 2007‐2014. B cell: B‐cell lymphoma, T/NK cell: T/NK‐cell lymphoma, Hodgkin: Hodgkin lymphoma, Others: other hematopoietic and lymphoid malignancies

**Figure 2:**
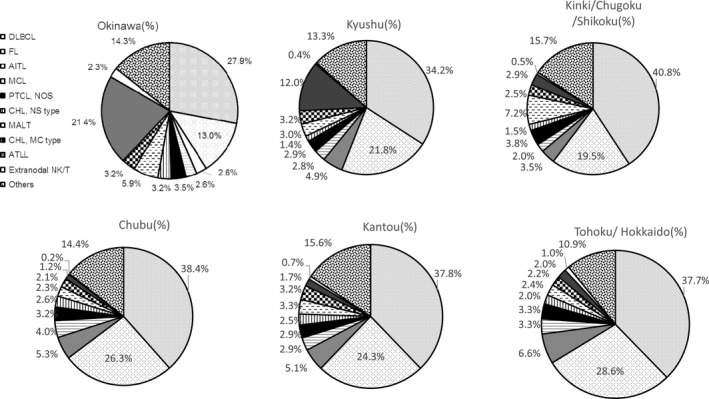
Frequency of sub‐major histological lymphoma subtypes in six geographic areas in Japan in 2007‐2014. DLBCL: diffuse large B‐cell lymphoma; FL: follicular lymphoma; AITL: angioimmunoblastic T‐cell lymphoma; MCL: mantle cell lymphoma; CHL, NS type: classic Hodgkin lymphoma, nodular sclerosis type; MALT: extranodal marginal zone lymphoma of mucosa‐associated lymphoid tissue; CHL, MC type: classic Hodgkin lymphoma, mixed cellularity

The histological subtypes of the malignant lymphomas in our study are listed in Table 1, and the distribution of each subtype among the nine geographic areas in Japan is presented in Table 1 and Figures 1 and 2. For the major subtypes, the overall percentages were as follows (with those for Okinawa, Kyushu, Kinki/Chugoku/Shikoku, Chubu, Kanto, and Tohoku/Hokkaido in parentheses): B‐cell lymphoma, 71.7% (54.6%, 68.6%,77.2%, 77.7%, 75.5%, and 76.9%, respectively); T/NK cell neoplasms, 18.5% (33.6%, 22.7%, 14.3%, 12.3%, 13.3%, and 14.8%, respectively); Hodgkin lymphoma, 5.8% (7.5%, 5.4%, 5.0%, 5.2%, 6.5%, and 4.4%, respectively); and histiocytic/dendritic cell neoplasms, 0.15% (0.39%, 0.12%, 0.09%, 0%, 0.22%, and 0%, respectively).

**Table 1:** Frequency of lymphoid neoplasms by subtype and geographical location/Age and sex distribution, 2007‐2014

	Median age	M/F	Japan (n)	Japan (%)	Okinawa (n)	Okinawa (%)	Kyushu (n)	Kyushu (%)	Kinki/Chu‐goku/Shikoku (n)	Kinki/Chugoku/Shikoku (%)	Chu‐bu (n)	Chubu (%)	Kantou (n)	Kantou (%)	Touhoku/Hokkaido (n)	Tohoku/Hokkaido (%)
Total cases(n)	68	1.1684695	9424	100.0	777	100	3420	100	1103	100	658	100	2765	100	697	100
B‐cell neoplasms	68	1.1	6759	71.72	424	54.6	2345	68.6	852	77.2	511	77.7	2088	75.5	536	76.9
T/NK‐cell neoplasms	70	1.4019337	1745	18.52	261	33.6	775	22.7	158	14.3	81	12.3	367	13.3	103	14.8
Hodgkin lymphoma	60	1.951087	543	5.76	58	7.5	183	5.4	55	5.0	34	5.2	181	6.5	31	4.4
Histiocytic/dendritic cell neoplasms	69	0.5555556	14	0.15	3	0.4	4	0.1	1	0.1	0	0.0	6	0.2	0	0.0
Composite lymphoma	71	0.8947368	72	0.76	7	0.9	21	0.6	7	0.6	10	1.5	20	0.7	7	1.0
Immunodeficiency‐associated lymphoproliferative disorders	69	0.6391753	159	1.69	10	1.3	51	1.5	14	1.3	12	1.8	61	2.2	11	1.6
EBV‐lymphoproliferative disorder	86	0.75	49	0.52	0	0.0	18	0.5	7	0.6	5	0.8	15	0.5	4	0.6
Malignant lymphoma, NOS	65	Male ONLY	3	0.03	0	0.0	1	0.0	1	0.1	0	0.0	1	0.0	0	0.0
Other hematopoietic neoplasms	63	2.2916667	80	0.85	14	1.8	22	0.6	8	0.7	5	0.8	26	0.9	5	0.7
B‐cell neoplasms																
Precursor B‐lymphoblastic leukemia/lymphoma	31	1	16	0.17	2	0.3	5	0.1	3	0.3	0	0.0	5	0.2	1	0.1
Plasmablastic lymphoma	77	3	12	0.13	2	0.3	7	0.2	1	0.1	1	0.2	1	0.0	0	0.0
CLL/SLL	68	1.3863636	105	1.11	5	0.6	39	1.1	20	1.8	5	0.8	29	1.0	7	1.0
Lymphoplasmacytic lymphoma	67	3.2	42	0.45	1	0.1	15	0.4	8	0.7	3	0.5	14	0.5	1	0.1
Mantle cell lymphoma	71	3.5689655	266	2.82	20	2.6	95	2.8	22	2.0	26	4.0	80	2.9	23	3.3
Follicular lymphoma	63	0.8957772	2110	22.39	101	13.0	747	21.8	215	19.5	173	26.3	673	24.3	199	28.6
Nodal marginal zone B‐cell lymphoma	70	0.8709677	58	0.62	3	0.4	13	0.4	9	0.8	6	0.9	20	0.7	7	1.0
Extranodal marginal zone B‐cell lymphoma (MALT)	67	0.6956522	351	3.72	46	5.9	104	3.0	79	7.2	15	2.3	90	3.3	17	2.4
Splenic marginal zone B‐cell lymphoma	69	1.3	23	0.24	0	0.0	8	0.2	3	0.3	3	0.5	9	0.3	0	0.0
Splenic b‐cell lymphoma/leukemia, unclassifiable	63	1.5	5	0.05	0	0.0	2	0.1	1	0.1	0	0.0	2	0.1	0	0.0
Hairy cell leukemia	64	Male ONLY	2	0.02	0	0.0	0	0.0	2	0.2	0	0.0	0	0.0	0	0.0
Plasma cell neoplasms	67	1	40	0.42	2	0.3	17	0.5	1	0.1	3	0.5	17	0.6	0	0.0
Diffuse large B‐cell lymphoma	72	1.136478	3397	36.05	217	27.9	1168	34.2	450	40.8	253	38.4	1045	37.8	263	37.7
Mediastinal large B‐cell lymphoma	40	1.9333333	44	0.47	3	0.4	19	0.6	5	0.5	6	0.9	10	0.4	1	0.1
Intravascular large B‐cell lymphoma	71	1	30	0.32	6	0.8	3	0.1	5	0.5	3	0.5	13	0.5	0	0.0
Pyothorax‐associated lymphoma	78	5	12	0.13	1	0.1	2	0.1	1	0.1	2	0.3	5	0.2	1	0.1
Burkitt lymphoma	71	2	66	0.70	7	0.9	31	0.9	4	0.4	6	0.9	16	0.6	2	0.3
B‐cell lymphoma, unclassifiable, with features intermediate between DLBCL and Burkitt lymphoma	70	1.0454545	134	1.42	11	1.4	53	1.5	12	1.1	8	1.2	41	1.5	9	1.3
B‐cell lymphoma, unclassifiable, with features intermediate between DLBCL and classical Hodgkin lymphoma	52	0.6666667	6	0.06	1	0.1	2	0.1	2	0.2	0	0.0	0	0.0	1	0.1
Lymphomatoid granulosis	80	2	3	0.03	0	0.0	1	0.0	0	0.0	1	0.2	1	0.0	0	0.0
B‐cell neoplasms‐not otherwise specified^a^	69	1.46	123	1.31	6	0.8	38	1.1	20	1.8	8	1.2	45	1.6	6	0.9
T/NK‐cell neoplasms																
T‐cell prolymphocytic leukemia	70	1.5	5	0.05	0	0.0	0	0.0	0	0.0	1	0.2	4	0.1	0	0.0
Precursor T‐lymphoblastic leukemia/lymphoma	30	2.625	58	0.62	3	0.4	21	0.6	4	0.4	3	0.5	24	0.9	3	0.4
Extranodal NK/T‐cell lymphoma, nasal type	62	1.2857143	64	0.68	18	2.3	13	0.4	6	0.5	1	0.2	19	0.7	7	1.0
MF/Sezary syndrome	64	2.8	19	0.20	2	0.3	5	0.1	6	0.5	0	0.0	6	0.2	0	0.0
MF	63	2.2	16	0.17	2	0.3	3	0.1	6	0.5	0	0.0	5	0.2	0	0.0
Sezary syndrome	71	Male ONLY	3	0.03	0	0.0	2	0.1	0	0.0	0	0.0	1	0.0	0	0.0
Angioimmunoblastic T‐lymphoma	72	1.2068966	448	4.75	20	2.6	168	4.9	39	3.5	35	5.3	140	5.1	46	6.6
Peripheral T‐cell lymphoma, not otherwise specified	70	1.9494949	294	3.12	27	3.5	100	2.9	42	3.8	21	3.2	81	2.9	23	3.3
Adult T‐cell leukemia/lymphoma	70	1.2196721	677	7.18	166	21.4	411	12.0	32	2.9	8	1.2	46	1.7	14	2.0
Anaplastic large cell lymphoma	59	2.1764706	109	1.16	11	1.4	43	1.3	13	1.2	9	1.4	25	0.9	8	1.1
ALK positive	38	2	39	0.41	3	0.4	22	0.6	3	0.3	1	0.2	6	0.2	4	0.6
ALK negative	66	2.2380952	69	0.73	8	1.0	21	0.6	10	0.9	8	1.2	18	0.7	4	0.6
Enteropathy‐type‐T‐cell lymphoma	66	0.8571429	13	0.14	1	0.1	7	0.2	1	0.1	0	0.0	4	0.1	0	0.0
Hepatosplenic T‐cell lymphoma	63	Male ONLY	2	0.02	0	0.0	0	0.0	1	0.1	0	0.0	1	0.0	0	0.0
Primary cutaneous CD30 positive T‐cell lymphoproliferative disorders	63	1.1666667	26	0.28	5	0.6	2	0.1	7	0.6	1	0.2	9	0.3	2	0.3
Primary cutaneous peripheral T‐cell lymphoma, rare subtype	62	1	22	0.23	6	0.8	4	0.1	6	0.5	0	0.0	6	0.2	0	0.0
Subcutaneous panniculitis‐like T‐cell lymphoma	62	1.5	5	0.05	2	0.3	0	0.0	1	0.1	2	0.3	0	0.0	0	0.0
T/NK‐cell lymphoma, not otherwise specified	33	Male ONLY	3	0.03	0	0.0	1	0.0	0	0.0	0	0.0	2	0.1	0	0.0
Hodgkin lymphoma																
Nodular LP Hodgkin lymphoma	41	2.6666667	11	0.12	2	0.3	2	0.1	1	0.1	0	0.0	5	0.2	1	0.1
Classical Hodgkin lymphoma																
NS classical Hodgkin lymphoma	38	1.2117647	188	1.99	25	3.2	47	1.4	17	1.5	17	2.6	68	2.5	14	2.0
MC classical Hodgkin lymphoma	64	2.6973684	281	2.98	25	3.2	110	3.2	28	2.5	14	2.1	89	3.2	15	2.2
LR classical Hodgkin lymphoma	60	3.5	8	0.08	2	0.3	1	0.0	3	0.3	1	0.2	1	0.0	0	0.0
LD classical Hodgkin lymphoma	70	2.5	35	0.37	1	0.1	16	0.5	5	0.5	2	0.3	10	0.4	1	0.1
Hodgkin lymphoma‐not otherwise specified	49	1.375	19	0.20	3	0.4	7	0.2	1	0.1	0	0.0	8	0.3	0	0.0
Histiocytic/dendritic cell neoplasms	69	0.5555556	14		3	0.4	4	0.1	1	0.1	0	0.0	6	0.2	0	0.0
Histiocytic sarcoma	68	0.8	7	0.07	1	0.1	2	0.1	1	0.1	0	0.0	3	0.1	0	0.0
Langerhans histiocytosis/sarcoma	71	0.4	7	0.07	2	0.3	2	0.1	0	0.0	0	0.0	3	0.1	0	0.0
Composite lymphoma	71	0.8947368	72	0.76	7	0.9	21	0.6	7	0.6	10	1.5	20	0.7	7	1.0
Immunodeficiency‐associated lymphoproliferative disorders																
Post‐transplant lymphoproliferative disorders	36	2	12	0.13	1	0.1	4	0.1	0	0.0	0	0.0	7	0.3	0	0.0
Other iatrogenic immunodeficiency associated lymphoproliferative disorders	69	0.5806452	147	1.56	9	1.2	47	1.4	14	1.3	12	1.8	54	2.0	11	1.6
MTX‐lymphoproliferative disorders	69	0.4941176	127	1.35	8	1.0	40	1.2	11	1.0	8	1.2	49	1.8	11	1.6
EBV‐lymphoproliferative disorder	86	0.75	49	0.52	0	0.0	18	0.5	7	0.6	5	0.8	15	0.5	4	0.6
Malignant lymphoma, not otherwise specified	65	Male ONLY	3	0.03	0	0.0	1	0.0	1	0.1	0	0.0	1	0.0	0	0.0
Other haematopoietic neoplasms																
Granulocytic sarcoma	61	2.2352941	55	0.58	10	1.3	16	0.5	6	0.5	5	0.8	14	0.5	4	0.6
Blastic plasmacytoid dendritic cell neoplasm	72	1.5	15	0.16	3	0.4	4	0.1	1	0.1	0	0.0	6	0.2	1	0.1
Multiple myeloma	66	2	8	0.08	0	0.0	2	0.1	1	0.1	0	0.0	5	0.2	0	0.0

Two cases of FL, 1 case of DLBCL and 1 case of Classical HL (LR type) lack geographical information. A case of ALCL lacks information about ALK expression information.

a B‐cell neoplasms‐not otherwise specified included 3 cases of high grade B‐cell lymphoma, nos and 95 cases of low grade B‐cell lymphoma, nos, etc

Overall, DLBCL is the most common and FL is the second in frequency followed by adult T‐cell leukemia/lymphoma (ATLL), AITL, MALT and PTCL, NOS.

**Figure 3:**
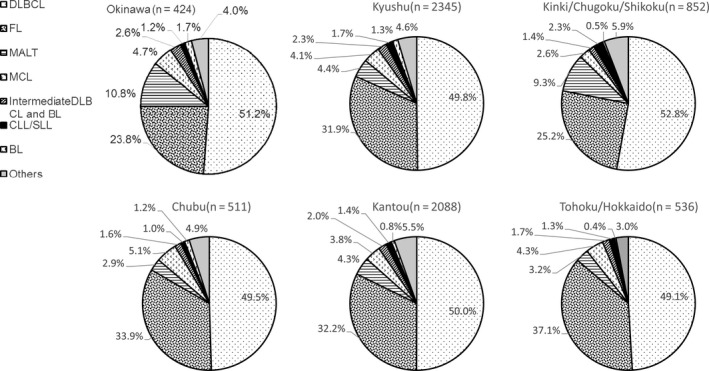
Frequency of B‐cell lymphoma subtypes in six geographic areas in Japan from 2007‐2014. DLBCL: diffuse large B‐cell lymphoma, FL: follicular lymphoma, MALT: extranodal marginal zone lymphoma of mucosa‐associated lymphoid tissue lymphoma, MCL: mantle cell lymphoma, BL: Burkitt lymphoma, CLL/SLL: chronic lymphoid leukemia/small lymphoid leukemia, Others: other B‐cell malignancies

Among B‐cell lymphoma, DLBCL was the most common (50.3%), followed by FL (31.2%), MALT (5.2%), and MCL (3.9%). This order also applied to the different regions, excepting Chubu and Tohoku/Hokkaido. In these two regions, MCL was more frequent than MALT lymphoma (Chubu: MALT, 2.9% and MCL, 5.1%; Tohoku/Hokkaido: MALT, 3.2% and MCL, 4.3%) (Figure 3).

**Figure 4:**
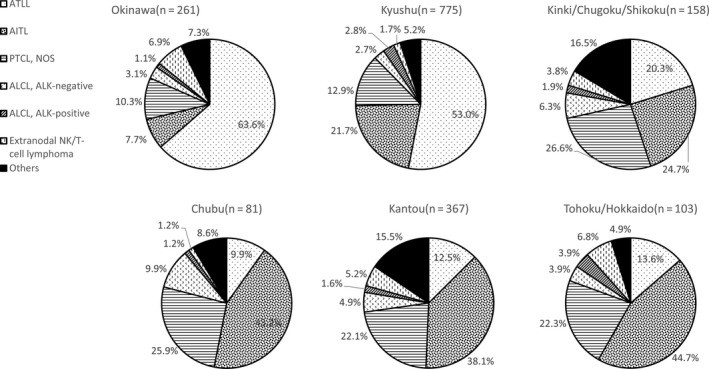
Frequency of T/NK cell lymphoma subtypes in six geographic areas in Japan from 2007‐2014. ATLL: adult T‐cell leukemia/lymphoma; AITL: angioimmunoblastic T‐cell lymphoma; PTCL‐NOS: peripheral T‐cell lymphoma, not otherwise specified; ACLC: anaplastic large cell lymphoma; ALK: anaplastic lymphoma kinase; NK: natural killer; Others: other T/NK‐cell malignancies

Among T/NK cell lymphomas, 38.8% were ATLL, 25.7.8% were AITL, 16.8% were PTCL‐NOS, and 6.2% were ALCL (2.2% ALK^＋^ and 4.0% ALK^−^). ATLL was the most common T/NK‐cell lymphoma in Kyushu (53.0%) and Okinawa (63.6%) (Figure 4). It was the third most frequent malignant lymphoma in Kyushu and the second most frequent malignant lymphoma in Okinawa; it ranked sixth ,13th, 11th,and ninth in Kinki/Chugoku/Shikoku, Chubu, Kanto, and Tohoku/Hokkaido, respectively (Figure 2). The proportion of extranodal NK/T‐cell lymphoma in Okinawa has declined significantly compared to that reported in a previous study by Aoki et al 2.3% (18 of 777 cases) in this study and 5.8% (17 of 292 cases) in the Aoki's study.^8^

### 3.2 Age distribution

Most of the patients in this study were >15 years old because patients ≤15 years old are almost exclusively diagnosed at the National Center for Child Health and Development in Japan. Accordingly, some types of lymphomas that occur in children may be elder than real age. Median ages at diagnosis were 68 years for B‐cell neoplasms, 70 years for T/NK‐cell neoplasms, 60 years for Hodgkin lymphoma, 69 years for histiocytic/dendritic cell neoplasms, 71 years for composite lymphoma, and 69 years for immunodeficiency‐associated lymphoproliferative disorder. Hence, with the exception of Hodgkin lymphoma, most major malignant lymphoma subtypes are diagnosed when patients are in their late 60s to 70s. The median and mean ages for the sub‐major subtypes were as follows: precursor B‐lymphoblastic leukemia/lymphoma, 31 and 32.06 years (n = 16); mediastinal B‐cell lymphoma, 40 and 49.16 years (n = 44); unclassifiable B‐cell lymphoma with features intermediate between those of DLBCL and classical Hodgkin lymphoma, 52 and 51.00 years (n = 6); precursor T‐lymphoblastic leukemia/lymphoma, 30 and 39.31 years (n = 58); ALK^−^ anaplastic large cell lymphoma, 38 and 39.44 years (n = 39); nodular lymphocyte predominant Hodgkin lymphoma, 41 and 47.27 years (n = 11); and the nodular sclerosis type of classical Hodgkin lymphoma, 38 and 43.92 years old, respectively (n = 188).

### 3.3 Sex distribution

**Figure 5:**
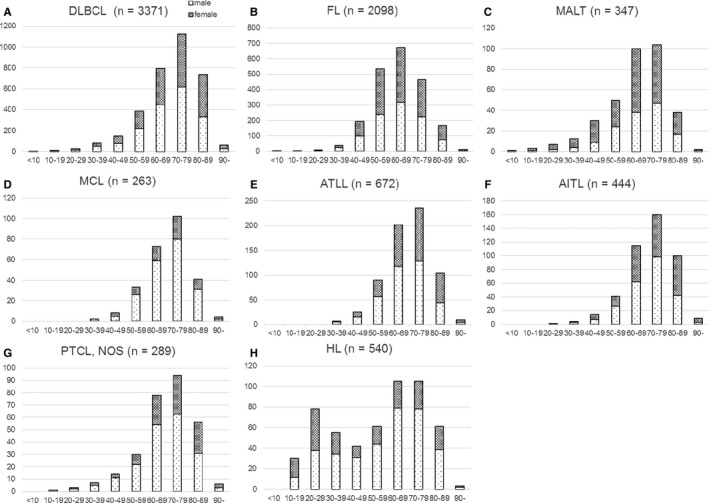
Number of representative lymphoid neoplasms according to age and sex in Japan from 2007‐2014. DLBLC: diffuse large B‐cell lymphoma; FL: follicular lymphoma; MALT: extranodal marginal zone lymphoma of mucosa‐associated lymphoid tissue; MCL: mantle cell lymphoma; ATLL: adult T‐cell leukemia/lymphoma; AITL: angioimmunoblastic T‐cell lymphoma; PTCL‐NOS: peripheral T‐cell lymphoma, not otherwise specified; HL: Hodgkin lymphoma

Similar to previous studies,^5^, ^8^ overall, the M/F ratio in our study was 1.17. Some subtypes showed male predominance (defined as an M/F ratio >3.0): MCL (M/F ratio, 3.57; n = 266), plasmablastic lymphoma (M/F ratio, 3.0; n = 12), lymphoblastic lymphoma (M/F, ratio 3.2; n = 42), and pyothorax‐associated lymphoma (M/F ratio, 5.0; n = 12). Only men had hairy cell leukemia (n = 2), Sezary syndrome (n = 3), hepatosplenic T‐cell lymphoma (n = 2), and malignant lymphoma not otherwise specified (n = 3); however, the number of cases were too small to refer to these as male‐predominant lymphomas. Only methotrexate‐associated lymphoproliferative disorders (MTX‐LPDs) showed female predominance (defined as an M/F ratio <0.5; M/F ratio, 0.49; n = 127). There were no significant differences in the M/F ratios for the major subtypes (1.14, 0.9, 1.22, 1.21, and 0.68 for DLBCL, FL, ATLL, AITL, and MALT, respectively) (Figure 5).

### 3.4 Secular trend of the age of onset for the major histological subtypes of malignant lymphoma

**Figure 6:**
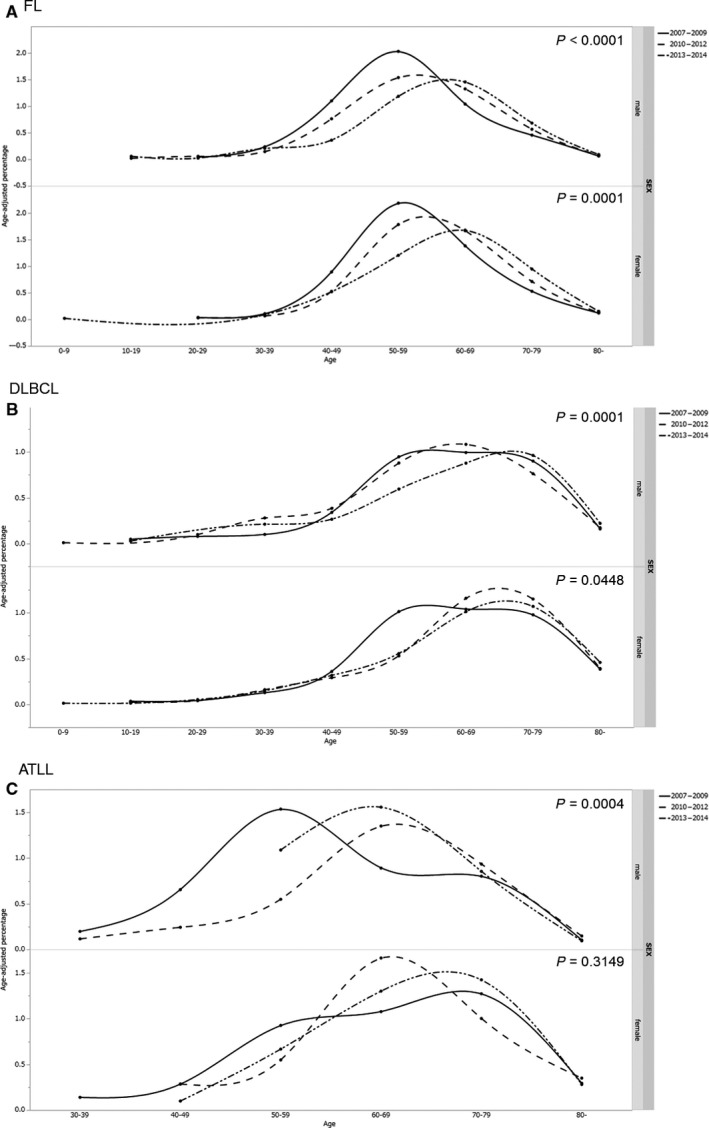
Secular trends in the onset age for FL, DLBCL, and ATLL. The top graph show significant differences in the onset age for FL (*P* < 0.0001) (A) DLBLC (*P* = 0.0001) (B) and ATLL (*P* = 0.0004) (C), across three time periods (2007‐2009, 2010‐2012, and 2013‐2014) in men. The bottom graph shows significant differences for FL (*P* = 0.0001) and DLBCL (*P* = 0.0448) but not ATLL (*P* = 0.3149) in women. ATLL: adult T‐cell leukemia/lymphoma, FL: follicular lymphoma, DLBCL: diffuse large B‐cell lymphoma

The age of onset for seven major subtypes (AITL, ATLL, DLBCL, FL, MALT, MCL, PTCL‐NOS) according to the time of diagnosis (2007‐2009, 2010‐2012, 2013‐2014) was determined. Onset age differed significantly across these time periods for ATLL (*P* = 0.0002), FL (*P* ≤ 0.0001), and DLBCL (*P* = 0.0002) only. Significant differences for ATLL, FL, and DLBCL were also apparent in analyses stratified for men (*P* = 0.0004, *P* < 0.0001, and *P* = 0.0001, respectively). As shown in Figure 6, significant differences were observed for FL (*P* = 0.0010) (Figure 6A) and DLBCL (*P* = 0.0448) (Figure 6B), but not ATLL (*P* = 0.3149) (Figure 6C), in analyses stratified for women.

## 4 DISCUSSION

Although most of the findings of this study confirmed those of previous reports,^7^, ^8^ some novel observations were made, as detailed below.

### 4.1 FL

**Figure 7:**
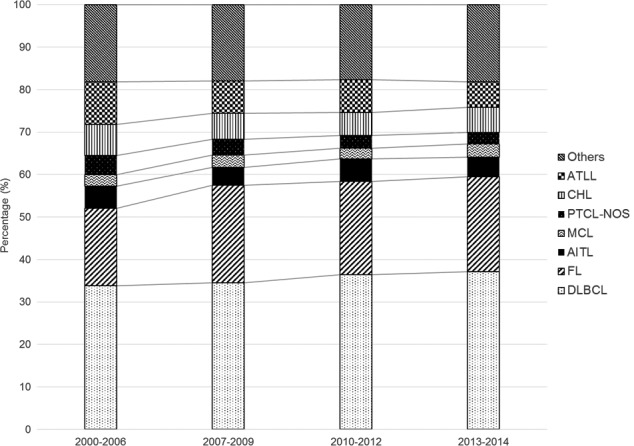
Secular trends in the frequency of representative histological lymphoma subtypes. A gradual increase in the proportion of FL is shown (18.3%, 23%, 21.9%, and 22.4% in 2000‐2006, 2007‐2009, 2010‐2012, and 2013‐2014, respectively). The percentages for 2000‐2006 were taken from the study by Aoki et al^8^ Others: other hematopoietic and lymphoid malignancies; ATLL: adult T‐cell leukemia/lymphoma; CHL: classic Hodgkin lymphoma; PTCL‐NOS: peripheral T‐cell lymphoma, not otherwise specified; MCL: mantle cell lymphoma; AITL: angioimmunoblastic T‐cell lymphoma; FL: follicular lymphoma; DLBCL: diffuse large B‐cell lymphoma

The proportion of FL is higher in Western countries (32% in Omaha, NE, USA; 31% in Vancouver, Canada; and 28% in London, UK) than in Asian regions (8% in Hong Kong, China).^2^ In Japan, it has gradually increased: 6% in 1996‐2000,^12^ 18.3% in 2000‐2006,8% and 22.4% in 2007‐2014 (the present study) (Figure 7). Hence, the proportion of FL in Japan is approaching that in Europe and the United States.

The onset age for FL in Asian countries is lower than that reported in international studies (59 years),^13^ with 54 years in southwest China,^14^ 52 years in eastern India,^15^ and 52.5 years in Korea.^16^ Our study shows a gradual increase in the median and mean age of patients with FL in Japan (*P* < 0.0001). These findings show that the onset age for FL is shifting from an Asian pattern to a Western pattern for reasons unknown.

### 4.2 DLBCL

Our study shows that onset age for DLBCL has increased in Japan (*P* = 0.0002) (Figure 6B). The median onset age for DLBCL in Japan was 72 years (mean age, 69.56 years), which is much higher than the median ages in Korea (59 years), 17 China (55 years),^14^ and internationally (64 years).^13^

The reason for the increasing onset age for DLBCL in Japan is unclear, but one hypothesis is worth noting. The proportion of secondary DLBCL, which tends to develop at a more advanced age than primary DLBCL, may be increasing. Due to a universal care system, elderly patients in Japan often receive chemotherapy, including molecular targeting therapy. Consequently, those with low‐grade malignant lymphomas survive longer, allowing more time for secondary DLBCL to arise.

In addition, although detailed risk factors of DLBCL have not yet been revealed, potential risk factors affecting the onset age for DLBCL include chronic inflammation^18^, ^19^ and an immunocompromised state due to advanced age,^20^ acquired immunodeficiency syndrome,^21^ primary immune disorders,^22^ certain medications,^23^ and transplants.^24^

### 4.3 ATLL

Adult T‐cell leukemia/lymphoma is caused by HTLV‐1, which is most prevalent in the southern regions of Japan, such as Kyushu and Okinawa.^25^ Hence, the geographical distribution of ATLL differs throughout Japan.

The onset age for ATLL differed significantly according to the year of diagnosis (2007‐2009, 2010‐2012, or 2013‐2014) (*P* = 0.002). Significant differences were also observed in analyses stratified for men (*P* = 0.0004), but not for women (*P* = 0.3149). There were no constant trends for both men and women (Figure 6C); in men, the onset age for ATLL was lowest in 2007‐2009, followed in order by 2013‐2014 and 2010‐2012. In women, onset age was lowest in 2010‐2012, followed in order by 2013‐2014 and 2007‐2009. Thus, although the differences between the time periods were statistically significant, we considered them to be essentially nonsignificant.

### 4.4 MTX‐LPD

**Figure 8:**
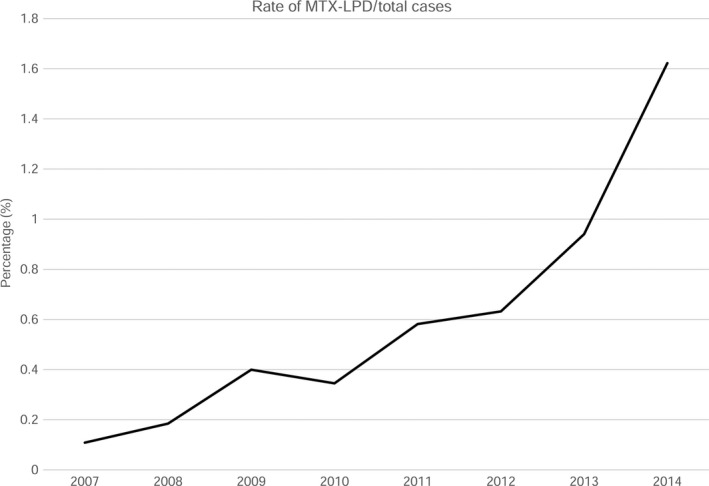
Frequency of MTX‐LPD cases per total hematopoietic and lymphoid tissue neoplasm cases from 2007‐2014. The frequency of MTX‐LPD cases was 0.11%, 0.18%, 0.40%, 0.35%, 0.58%, 0.63%, 0.94%, and 1.6% in 2011‐2014, respectively. MTX‐LPD: methotrexate‐associated lymphoproliferative disorder

Methotrexate‐associated lymphoproliferative disorder is an “other iatrogenic immunodeficiency‐associated lymphoproliferative disorder” that occurs in patients treated with MTX.^5^ In this study, the median age at which MTX‐LPD was diagnosed was 69 years, and the M/F ratio was 0.49. This female predilection presumably reflects the frequency of autoimmune diseases in women, including rheumatoid arthritis (RA).^26^ The number of MTX‐LPD cases has increased since 2008 (Figure 8). Two events may account for this increase: first, its inclusion in the 4th edition of the WHO guidelines in 2008, which widened its recognition by clinicians and second, the increased use of MTX. MTX was indicated for rheumatoid arthritis in 1999 and become the drug of first alternative in 2011 in Japan.

### 4.5 Strengths and limitations

The strengths of our study are as follows. First, to the best of our knowledge, it is the largest epidemiological analysis performed in Japan, with >9000 cases. Second, all cases were diagnosed by the same pathologist, thus eliminating the need for resolution of diagnostic differences. Third, because Japan consists of essentially one race, consideration of racial factors was unnecessary.

Our study did, however, have limitations. First, most of the specimens were submitted to us by hematologists. Because some malignant lymphomas (eg, MALT lymphomas) are also treated by gastroenterologists, ophthalmologists, and dermatologists, their frequencies may be greater than reported here. Second, the frequency of subtypes usually diagnosed via analysis of peripheral blood (eg, hairy cell leukemia, CLL/SLL, and acute types of ATLL) may have been underestimated because blood samples were not submitted to us. Third, because ATLL has several histological forms, measurement of HTLV‐1 antibody levels and detection of monoclonal integration of the HTLV‐1 provirus are necessary for an accurate diagnosis.^27^ However, these assays were probably not performed in regions in which HTLV‐1 is not endemic, potentially resulting in underestimation of ATLL frequency. Fourth, as noted above, patients ≤15 years old in Japan are almost exclusively diagnosed in a hospital specializing in pediatrics. Therefore, the true epidemiology of some lymphoma subtypes may not be reflected in our study. Fifth, although we received data for this study from throughout Japan, the proportion of data received from Kyushu and Okinawa was relatively high. Thus, there is a possible bias caused by geographical distribution. Table 2 shows a comparison between the present study and related epidemiological studies in Japan. Although the present study included many specimens from Kyushu and Okinawa, which reportedly have high numbers of ATLL cases, Table 2 shows that the proportion of T‐cell lymphomas was relative low compared to that reported in previous studies. The reason for this is unclear, but it may be due to an increased proportion of FL and DLBCL. Finally, knowledge of a patient’s history of MTX therapy is essential for diagnosis of MTX‐LPD. However, the accuracy of such information may vary from hospital to hospital. In Japan, MTX is often prescribed by orthopedic specialists and rheumatologists. Large hospitals have many departments (eg, Orthopedics and Rheumatology) that share information about MTX administration, and thus, this information is likely reliable. On the other hand, in clinics or small hospitals, information about MTX use may not be readily available because MTX may have been prescribed elsewhere. Thus, the frequency of MTX‐LPD in small hospitals and clinics may be underestimated.

**Table 2:** Comparison with related epidemiological studies in Japan

	Current study (2007‐2014) (n = 9424) (%)	LSG of Japanese pathologist (1994‐1996) (n = 3194) (%)	Chihara et al (2003‐2008) (n = 125148) (%)
B‐cell neoplasms	71.72	68.53	
T/NK‐cell neoplasms	18.52	24.92	
Hodgkin lymphoma	5.76	4.41	5.90
Histiocytic/dendritic cell neoplasms	0.15	0.31	
Other hematopoietic neoplasms	3.85		
B‐cell neoplasms			
Precursor B‐lymphoblastic leukemia/lymphoma	0.17	2.35	
Plasmablastic lymphoma	0.13		
CLL/SLL	1.11	1.31	3.20
Lymphoplasmacytic lymphoma	0.45	0.69	
Mantle cell lymphoma	2.82	2.79	2.0
Follicular lymphoma	22.39	6.70	13.50
Nodal marginal zone B‐cell lymphoma	0.62	1.00	
Extranodal marginal zone B‐cell lymphoma (MALT)	3.72	8.45	
Splenic marginal zone B‐cell lymphoma	0.24	0.13	
Hairy cell leukemia	0.02	0.16	
Plasma cell neoplasms	0.42	1.10	
Diffuse large B‐cell lymphoma	36.05	33.34	45.30
Mediastinal large B‐cell lymphoma	0.47	0.25	
Intravascular large B‐cell lymphoma	0.32	0.09	
Pyothorax‐associated lymphoma	0.13	0.28	
Burkitt lymphoma	0.70	1.00	1.30
Other B‐cell lymphomas	2.88		
T/NK‐cell neoplasms			
T‐cell prolymphocytic leukemia	0.05	0.06	
Precursor T‐lymphoblastic leukemia/lymphoma	0.62	1.72	
Extranodal NK/T‐cell lymphoma, nasal type	0.68	2.60	
MF/Sezary syndrome	0.20	1.16	
MF	0.17		1.0
Sezary syndrome	0.03		
Angioimmunoblastic T‐lymphoma	4.75	2.35	2.0
Peripheral T‐cell lymphoma, not otherwise specified	3.12	6.67	4.10
Adult T‐cell leukemia/lymphoma	7.18	7.45	8.30
Anaplastic large cell lymphoma	1.16	1.53	1.10
Enteropathy‐type‐T‐cell lymphoma	0.14	0.25	
Hepatosplenic T‐cell lymphoma	0.02	0.06	
Primary cutaneous CD30 positive T‐cell lymphoproliferative disorders	0.28	0.25	
Subcutaneous panniculitis‐like T‐cell lymphoma	0.05	0.06	
Other T/NK‐cell lymphomas	0.27		
Hodgkin lymphoma			
Nodular LP Hodgkin lymphoma	0.12	0.16	
Classical Hodgkin lymphoma			
NS classical Hodgkin lymphoma	1.99	1.78	
MC classical Hodgkin lymphoma	2.98	1.63	
LR classical Hodgkin lymphoma	0.08	0.25	
LD classical Hodgkin lymphoma	0.37	0.25	
Hodgkin lymphoma‐not otherwise specified	0.20		

## 5 CONCLUSION

This study shows that the proportion of FL in Japan has increased between 2007 and 2014, as has the onset age for FL and DLBCL. Future monitoring of this trend should continue in the future, and the reasons for these increases should be determined.

## CONFLICT OF INTEREST

None declared.
